# Gut Microbiome–Brain Axis as an Explanation for the Risk of Poor Neurodevelopment Outcome in Preterm Infants with Necrotizing Enterocolitis

**DOI:** 10.3390/microorganisms11041035

**Published:** 2023-04-15

**Authors:** Jason Xia, Erika C. Claud

**Affiliations:** 1 College of Liberal Arts and Sciences, University of Illinois Urbana-Champion, Champaign, IL 61801, USA; 2 Department of Pediatrics and Medicine, The University of Chicago, Chicago, IL 60637, USA

**Keywords:** necrotizing enterocolitis, brain, gut–brain axis, gut microbiota–brain (GMB) axis, intestinal inflammation, metabolite, microbiome, insulin growth factor 1, blood–brain barrier

## Abstract

Necrotizing Enterocolitis (NEC) is characterized by an inflammation of intestinal tissue that primarily affects premature infants. It is the most common and devastating gastrointestinal morbidity of prematurity, but beyond intestinal morbidity, this condition has also been associated with an increased risk of neurodevelopmental delays that persist beyond infancy. Prematurity, enteral feeding, bacterial colonization, and prolonged exposure to antibiotics are all risk factors that predispose preterm infants to NEC. Interestingly, these factors are all also associated with the gut microbiome. However, whether or not there is a connection between the microbiome and the risk of neurodevelopmental delays in infants after NEC is still an emerging area of research. Furthermore, how microbes in the gut could impact a distant organ such as the brain is also poorly understood. In this review, we discuss the current understanding of NEC and the role of the gut microbiome–brain axis in neurodevelopmental outcomes after NEC. Understanding the potential role of the microbiome in neurodevelopmental outcomes is important as the microbiome is modifiable and thus offers the hope of improved therapeutic options. We highlight the progress and limitations in this field. Insights into the gut microbiome–brain axis may offer potential therapeutic approaches to improve the long-term outcomes of premature infants.

## 1. Introduction

Necrotizing Enterocolitis (NEC) is an inflammatory bowel condition that primarily affects premature infants. Although NEC is classically described in terms of intestinal signs and symptoms, it is known to have systemic effects and consequences. This condition is characterized not only by inflammation of the intestine but also by increased risk of neurodevelopmental delays. As research continues and progresses, our understanding of NEC continues to expand; however, much remains unknown, and treatment options are still limited.

In recent years, research has emphasized the role of the communication axis between the gut and the nervous system, the gut–brain axis [1,2], as well as the establishment of the importance of the microbiome in both gut and brain signaling. Interestingly, recent data have provided a connection between the early gut microbiota colonization of infants and short- and long-term health implications. This review serves to highlight and discuss the current understanding of NEC and the role of the gut microbiome–brain axis in neurodevelopmental outcomes after NEC. A brief overview of NEC and the known neurodevelopmental consequences associated with it are given, followed by the potential factors that contribute to neurodevelopmental risk including inflammatory cytokines, the microbiome, and the gut microbiome–brain axis, as well as the means by which these factors can be altered to prevent or treat NEC.

## 2. Neonatal Necrotizing Enterocolitis (NEC)

NEC is an inflammatory intestinal condition that affects approximately 5–7% of preterm infants born at <1500 gm [3,4]. Typical initial signs and symptoms of the condition include feeding intolerance, abdominal distention, and bloody stools after 8 to 10 days of age [5,6]. Beyond intestinal symptoms, NEC is also associated with systemic signs of hypotension, an increased need for respiratory support, and electrolyte abnormalities. If not treated and detected early, symptoms may rapidly progress to intestinal perforation, peritonitis, and systemic shock with high morbidity and mortality [5]. Current treatments for NEC include both medical and surgical interventions. Medical treatment is primarily non-specific supportive care including bowel rest, broad-spectrum antibiotics, parenteral nutrition, correction of electrolyte abnormalities, respiratory support, and close monitoring for progression of the disease. Infants who progress to intestinal perforation or have evidence of ongoing necrosis require surgical intervention. Surgical interventions include both laparotomy and primary peritoneal drainage [7], with recent studies, particularly the Necrotizing Enterocolitis Surgery Trial (NEST) trial, highlighting the importance of distinguishing between NEC and spontaneous intestinal perforation [8] for therapeutic and surgical decisions. Due to the frequent rapid progression of NEC from the time of diagnosis, it has been suggested that prevention is key. Studies have indicated that breast milk feeding and antibiotic stewardship are important means of decreasing NEC incidence [9,10].

## 3. Neurodevelopmental Risk Associated with Necrotizing Enterocolitis

In addition to its gastrointestinal impacts, NEC, particularly surgical NEC, is an independent risk factor for long-term neurodevelopmental impairments in infants. A landmark study by the National Institute of Child Health and Human Development Neonatal Research Network Registry analyzed 2498 infants who had neurodevelopmental evaluation at 18–22 months to compare outcomes among infants who had NEC requiring surgery, infants who had NEC requiring medical management only, and those who did not have NEC. Infants who required surgery to treat NEC were more likely to have poor growth and cystic periventricular leukomalacia [11]. They were also more likely to have abnormal cognitive and motor development and to be at higher risk for any neurodevelopmental impairment than infants without NEC [1,11,12,13,14]. There are many potential explanations for the increased risk of neurodevelopmental impairment. Fluctuations in blood pressure or blood oxygen levels associated with the systemic manifestations of severe NEC may increase neurodevelopmental risk. Suboptimal protein delivery with prolonged parenteral rather than enteral nutrition during bowel rest may be insufficient for the needs of brain development. Anesthesia associated with surgical procedures has been associated with adverse effects on the developing brain. It has specifically been shown that at term-corrected gestational age, preterm infants exposed to general anesthesia have smaller white matter volumes observed by magnetic resonance imaging (MRI) and delayed neurodevelopment measured by the Bayley Scales of Infant and Toddler Development, and animal studies have indicated that anesthesia is associated with apoptosis of neurons [15]. Additionally, the inflammation associated with NEC is not confined to the intestine. Studies have documented increased levels of inflammatory cytokines including interleukin (IL)-1β, IL-6, and IL-8 in serum samples of infants with NEC that may directly reach the developing preterm brain with adverse consequences [16]. 

## 4. Inflammatory Cytokines in Intestinal and Brain Injury Associated with Necrotizing Enterocolitis

The immature intestine has an exaggerated inflammatory response. Studies in human immature intestinal epithelial cell lines have demonstrated increased IL-8 secretion in response to both commensal and pathogenic bacteria associated with decreased inhibition of the transcription factor nuclear factor kappa B (NF-κB) [17]. IL-8 is a chemokine known to activate neutrophils leading to intestinal necrosis and increased production of acute-phase proteins in the gut. Increases in IL-8 as well as other NF-κB-dependent cytokines and chemokines may predispose the immature intestine of preterm infants to NEC because it expects a sterile intrauterine environment and may be ill-prepared for the excessive microbial stimulation of postnatal colonization. Clinical studies, furthermore, have shown that the serum levels of several cytokines and chemokines including IL-1β, IL-6, IL-8, monocyte chemoattractant protein-1/CC-motif ligand (CCL)-2, macrophage inflammatory protein-1β/CCL3, and C-reactive protein are higher in those with NEC compared with those without [16].

Studies in animal models have begun to shed light on the impact of systemic inflammatory cytokines on potential brain injury associated with NEC. In an animal model of NEC, compared with normal rats, pathological damage to periventricular white matter was observed in the NEC group. This mirrors the increased risk of cystic perventricular leukomalacia in surgical NEC patients identified in the previously mentioned NICHD study [11]. In this study, rats were given 3% dextran sulfate sodium to induce intestinal injury. The results demonstrated that brain white matter injury was associated with increases in the proinflammatory CXC chemokines CXCL1 and CXCR2 [18]. Additionally, a model in which mouse pups were stressed with hyperosmolar formula, hypoxia, and lipopolysaccharide (LPS) to induce NEC demonstrated that pups exposed to NEC stress had smaller brains associated with increased apoptosis, decreased numbers of neurons, and increased levels of the proinflammatory mediators IL-6 and tumor necrosis factor alpha [19]. Interestingly, mouse models of maternal intrauterine infection, which is associated with preterm birth, have demonstrated increased permeability of the blood–brain barrier in exposed pups, which may explain the increased exposure of the brain to systemic inflammatory cytokines in preterm infants [20].

Connections exist among hypoxia–ischemia, nutrition, infection, gut inflammation insults, and the developing brain in NEC [21], and a multifactorial explanation is likely. Recent studies suggest a role for the microbiome in both the development of NEC and neurodevelopmental outcomes as a potential common mediator of these multiple factors.

## 5. The Microbiome and Necrotizing Enterocolitis

The microbiome is the community of microorganisms including bacteria, fungi, viruses, and archaea that reside in or on the human body. The majority of these organisms are within the digestive tract of the host as the gut microbiome. The gut microbiome impacts both metabolism and immune function and has been shown to be a key player in health and disease [22,23]. Importantly for infants, the gut microbiome influences development. Preterm infants are unique, as they complete development that would normally occur in the sterile intrauterine environment in the hospital environment of the neonatal intensive care unit. The microbiome is known to be affected by factors such as age, diet, and medications, and as such is a mediator of the impact of the environment on human physiology. Thus, for the preterm infant, the unique environmental factors of the neonatal intensive care unit such as instrumentation, frequent antibiotics, potential long periods without enteral feeds, high-concentration oxygen delivery, separation from parents, multiple hospital caregivers, and exposure to hospital bacteria with antibiotic resistance genes have the potential to impact microbiome development and thus the physiology and development of the preterm infant as well [24].

Prematurity, enteral feeding, and bacterial colonization are all risk factors that predispose preterm infants to NEC. Breast feeding in particular has been shown to be protective against NEC and associated with many beneficial factors found in mother’s own milk including growth factors, human milk oligosaccharides, and immunoglobulin A [10,25,26,27]. Studies have even shown a dose response to the protective benefit of mother’s own milk for NEC in the first 14 days of life [10]. In addition, prolonged exposure to antibiotics has been associated with an increased risk of NEC among very-low-birth-weight infants. Cotten et al. demonstrated that prolonged empiric antibiotics immediately after birth was associated with increased risk of NEC and death, with risk increasing for each day of prolonged antibiotics [9]. In other studies, the risk of NEC has been shown to be associated with specific antibiotics. Gentamicin and meropenem, but not other antibiotics, are described as having a significant association with the incidence of NEC [28]. These risk factors for NEC are also associated with alterations in the microbiome.

The gut microbiome is of great relevance to NEC. The ability to deeply interrogate the intestinal microbiome with non-culture-based genomic sequencing technology has given us tools to better understand NEC pathogenesis and perhaps develop therapies for treatment and prevention [22]. Several studies have analyzed fecal microbiota from preterm infants with and without NEC [23,29]. These studies have found an overall decrease in diversity in the microbiome and changes in microbial species [22,23,29]. The increased microbiome diversity in preterm infants without NEC may serve as a protective measure against pathogens which cause intestinal inflammation. This protection may occur by competing for nutrients or receptors, or by enhancing development of the innate immune system [5]. There appears to be an association between an alteration in diversity with a bloom of *Gammaproteobacteria*, specifically *Enterobacteriaceae*, and an increased risk of NEC [29,30,31]. Conversely, multiple studies have demonstrated a decreased incidence of NEC with probiotic treatment, most commonly *Bifidobacteria* and *Lactobacillus* species [32,33,34,35]. Probiotics are any microorganism that has a health benefit beyond nutrition. Factors in breast milk, specifically human milk oligosaccharides, promote the growth of beneficial organisms such as *Bifidobacteria* and may explain part of the beneficial effect of breast milk feeding [36]. These factors which promote the growth of beneficial bacteria are termed prebiotics.

Bacterial colonization is a key risk factor for NEC, but not in the classical sense of an infection. It is thought to be due to the increased responses of the immature preterm intestine to bacterial products. This is associated with increased levels of toll-like receptor 4 (TLR 4) on the immature intestinal epithelium and endothelium. TLR 4 is a receptor for the bacterial surface motif LPS from gram-negative bacteria. Binding of TLR 4 leads to the activation of the NF-κB pathway and the transcription of multiple genes involved in key cellular processes. TLR 4 can lead to intestinal epithelial apoptosis, increased inflammatory cytokine production, and decreased intestinal blood flow when activated by the microbiome after birth [37]. However, TLR 4 is also important for normal intestinal development in utero and thus has beneficial roles as well [37].

This is just one of many lines of evidence suggesting that the interactions between microbes and the gut are not just pathogenic and inflammatory. In fact, an alternative hypothesis suggests that it is not just that dysbiosis is associated with increased pathogens that predispose infants to NEC, but that dysbiosis is associated with a failure of intestinal maturation which is critical to protection against NEC. Many elements of intestinal immaturity are thought to predispose preterm infants to NEC including altered surface glycoconjugate patterns, delayed motility, diminished numbers of paneth cells and levels of defensins, immature tight junction patterns, decreased numbers of goblet cells and levels of intestinal mucus, decreased levels of immunoglobulins, and increased activation of NF-κB-dependent responses [37]. All of these together increase the immature gut’s interaction with and responses to commensal microbes. These interactions result in inflammatory mediators and metabolites that are produced by the interaction of gut microbes with dietary substrates but can reach the systemic circulation to have distant effects. It has also been shown that certain preterm infant microbiome communities are associated with improved intestinal barrier tight junction function and decreased inflammation—both locally at the intestine but also systemically [38]. Therefore, the microbiome can functionally impact which microbial-mediated factors reach the systemic circulation and how readily they reach the systemic circulation to influence other organs. Specifically relevant to neurodevelopmental outcomes, studies have demonstrated that altering the microbiome can influence blood–brain barrier maturation and thus the interaction of the microbiome with the brain [20]. 

## 6. Gut Microbiome–Brain Axis in Necrotizing Enterocolitis

The gut microbiome regulates a relationship with the brain known as the gut microbiota–brain (GMB) axis. This bidirectional communication between the brain and the microbes residing in the gut comprises the enteric nervous system, the vagus nerve, immune factors, hormones, and bacterial metabolites such as neurotransmitters and short-chain fatty acids [39,40,41]. The GMB axis has been associated with childhood neurodevelopment disorders such as attention-deficit hyperactivity disorder (ADHD) and autism and with cognitive decline in older adult populations [42,43,44,45]. However, recent studies have also highlighted the role of the gut microbiome in infant development including brain development [46]. Disruptions and shifts in microbiome development that result in intestinal dysbiosis leading to NEC may thus also have a direct influence on brain development [41,47,48].

In NEC survivors, neurodevelopmental impairment is frequently seen and is believed to be correlated with the severity and extent of the NEC [49]. A study by Zhou and colleagues highlights this connection by demonstrating that brain samples from neonatal mice or human infants with NEC have increased numbers of CD4^+^ T cells. Extensive studies in mouse models suggest that these CD4^+^ T cells originate in the gut and secrete interferon gamma (IFNγ) resulting in the activation of brain microglia and induced injury [2,50]. The precise antigen responsible for activating the T cells is unknown, but the gut origin of the T cells suggests that a microbiome influence is possible [37,50].

There are interesting parallel windows of preterm infant microbiome development and brain development that may also impact outcomes. Preterm birth deprives preterm infants of a critical period of normal brain development and maturation *in utero*, since fundamental processes such as cortical and grey matter volumetric growth, neurogenesis, axonal and dendritic growth, synaptogenesis, and myelination begin in utero as early as 20 weeks’ gestation and are later pruned and modified during early postnatal development [51]. For the preterm infant, the postnatal time point occurs much earlier in development so that processes that would normally occur in the relatively sterile intrauterine environment now occur in the context of the extra-uterine environment and the microbiome.

The infant microbiome undergoes sequential development after birth termed succession. For preterm infants, this progression is also different from what occurs for full-term infants with rapid changes in the first few weeks of life informed by a hospital environment. Our previous studies demonstrated clustering of microbial composition patterns at two weeks of age and three to five weeks of age, followed by a progression that converged on full-term infant patterns only after six weeks of age [47]. The intersection of microbiome developmental stage and brain developmental stage likely impacts the functional neurodevelopment. Since microbiome patterns have also been shown to impact intestinal development and susceptibility to NEC, these altered microbiome patterns in infants with NEC may result in a simultaneous increased risk of neurodevelopmental compromise.

To examine whether different microbiota colonization patterns have an impact on early neuronal development, our group used a mouse model to measure the expression levels of early development markers in the brain in the context of different microbiome communities. We used a gnotobiotic mouse model in which human infant microbiome communities were transfaunated to germ-free mouse dams and passed on to mouse pups to allow assessment of tissue level effects with a human clinical context [52]. The fecal samples used were from human preterm infants with two different growth rates during their neonatal intensive care unit course, associated with differences in intestinal inflammatory profiles. Pups born to dams colonized with the microbiome of an infant with good growth had low intestinal inflammatory marker profiles, while pups born to dams colonized with the microbiome of an infant with poor growth had high intestinal inflammatory marker profiles [52]. Interestingly, Western blot analysis of cerebral cortex homogenates with a marker of neuron number (anti-NeuN antibody) showed significantly increased levels of NeuN expression in mice colonized with microbiota from a preterm infant with good growth compared with pups colonized with microbiota from a preterm donor with poor growth [12]. There was also evidence of increased myelination in the pups with the high-growth/low-intestinal-inflammation microbiome compared with the low-growth/high-intestinal-inflammation microbiome [12].

Our data also suggest that preterm microbiota may mediate brain development through the insulin growth factor 1 (IGF 1) pathway. Previous studies have shown that mutation(s) in the *igf*-1 gene or in the *igf1r* gene are found to be associated with severe body growth failure, microcephaly, and developmental delay [53]. In rodents, *igf-1* gene disruption results in reduced brain size and hypomyelination [53]. Furthermore, IGF-1 crosses the blood–brain barrier, and germ-free mice without any microbiome have been shown to have lower circulating IGF-1 levels compared with normally colonized specific-pathogen-free (SPF) mice, also suggesting a role for the microbiome [54,55]. Colonization of germ-free mice with human fecal samples from the preterm infant with poor growth was associated with decreased circulating and brain IGF-1 levels. However, since there were no differences in brain *Igf1* or *Igfr1* mRNA levels among the three experimental groups, the brain differences were most likely associated with serum differences resulting in different levels crossing the blood–brain barrier [12].

Additionally, serial microbiome samples from a large cohort of preterm infants found that different microbiome patterns were associated with differences in head circumference growth. Head circumference is a proxy for brain growth in an infant and has been correlated with long-term neurodevelopmental outcomes [56]. Differences in *Bacteriodota*, *Lachnospiraceae*, and *Actinobacteriota* were associated with differences in head circumference growth [46]. In term infants *Bacteroides* and *Lachnospiraceae* have been associated with functional brain connectivity [57]. It may be that a loss of *Bacteriodota*, *Lachnospiraceae*, and *Actinobacteriota* associated with a relative increase in *Gammaproteobacteria* predisposes infants to NEC as well as poor brain development [46].

Beyond identifying microbes by name and taxonomy, it is important to understand the function of the microbes and how they impact the host. Metagenomic analysis of fecal samples from infants that went on to develop NEC compared with control preterm infants that did not develop NEC identified differences in pathways associated with carbohydrate metabolism, antibiotic resistance, and vitamin biosynthesis [47]. A key means by which the microbiome interacts with the host is the production of metabolites from the breakdown of dietary substrates.

Metabonomics, or the study of the metabolites produced by a community of organisms, is an emerging research area with potential for improving our understanding of NEC and developing novel biomarkers for NEC risk. Microbiome-associated metabolites may also represent a possible tool for the prevention and treatment of the neurological affects connected with NEC; however, to date there has been no clear pattern observed [44,58,59]. A large prospective multicenter case–control study compared a targeted fecal metabolomics panel of amino acids and alcohols from infants 1–3 days before NEC with control preterm infants. Targeted high-performance liquid chromatography found increases in isoleucine, leucine, methionine, phenylalanine, and valine and decreases in lysine and ethanolamine that resulted in a model with a moderate ability to predict NEC [60]. A longitudinal study comparing metabolites in urine samples of NEC preterm infants compared with a control group without NEC used proton nuclear magnetic resonance spectroscopy (^1^H NMR) to find that late-onset NEC was associated with high lactate and decreased betaine, creatine, urea, myo-inositol, and *N*,*N*-dimethylglycine [61]. An animal study using preterm pigs as a model for NEC found higher serum levels of alanine, histidine, and *myo*-inositol and lower levels of 3-hydroxybutyric acid and isobutyric acid in animals with NEC [62]. Targeted metabolomics analysis of NEC and control fecal samples found that fecal fomic acid (fomate) was significantly increased at NEC onset and decreased with recovery [63]. Further experiments in a mouse model of NEC found that fomate induced intestinal injury [63]. Other studies have shown that the short-chain fatty acid butyrate, that is frequently cited as a beneficial metabolite in other contexts, specifically induces injury in immature intestinal cells [64].

Due to the variety of metabolites and their local and systemic effects, metabolites which are altered systemically may be a key element for gut–brain axis communication [40]. Short-chain fatty acids, tryptophan metabolites, and biliary acids may influence brain development and function through their regulation of immune cell pathways and the subsequent effect on the central nervous system [65]. Notably, short-chain fatty acids have been shown to directly and indirectly impact the gut–brain axis by regulating different immune, endocrine, epigenetic, and humoral mechanisms. 

Recent studies have focused on the blood–brain barrier as a key intersection between the systemic circulation and the brain for the interaction of molecules such as metabolites. The blood–brain barrier begins to form *in utero* and is a carefully regulated gatekeeper that allows nutrients but prevents bacteria and potentially harmful molecules from crossing from the blood to the brain. Capillary endothelial cells connected with tight junctions and joined by the addition of astrocytes and pericytes form the blood–brain barrier over time [66]. This process is incomplete when a preterm infant is born and begins contact with the microbiome, potentially increasing interactions between the developing preterm infant brain and bacteria and bacterial metabolites. Studies have shown that germ-free mice have prolonged increased permeability of the blood–brain barrier associated with decreased expression of the specific tight junction proteins Occludin and Claudin-5 [66]. Other studies have shown that inflammation, associated with increased systemic inflammatory cytokines as seen in NEC, further increases blood–brain barrier permeability [67]. Correspondingly, the probiotics *Lactobacillus acidophilus* and *Bifidobacterium infantis*, the same probiotics that have been shown to decrease the incidence of NEC, improve the blood–brain barrier specifically by increasing the tight junction protein Occludin [67]. Thus, the microbiome also has a role in regulating the blood–brain barrier itself.

## 7. Prevention and Therapy

Current treatments for NEC include both medical and surgical interventions. Medical treatment includes bowel rest, broad-spectrum antibiotics, parenteral nutrition, correction of electrolyte abnormalities, respiratory support, and close monitoring for progression of the disease. Despite a lack of complete understanding of the mechanism of NEC pathogenesis, the microbiome and the understanding of its role in NEC development has led to the microbiome being a possible area of interest for prevention and treatment [68]. The means of altering the microbiome in beneficial ways which have been shown to decrease the incidence of NEC and thus may prevent NEC include breastfeeding, antibiotic stewardship, and the use of probiotics [9,68,69,70,71]. However, there is still risk associated with the use of probiotics due to the immature immune systems of preterm infants. This immaturity leaves them vulnerable to even beneficial bacteria; therefore, further research is required to investigate both the short- and long-term effects of probiotic administration to preterm infants [72]. The Committee of the Fetus and Newborn of the American Academy of Pediatrics specifically does not support the routine administration of probiotics to preterm infants given the current state of data and available products [73]. While the supposition has been that NEC’s associated inflammation or nutrition alterations lead to NEC, it is possible that a common microbiome pattern may predispose infants to both NEC and altered brain development.

## 8. Conclusions

**Figure 1:**
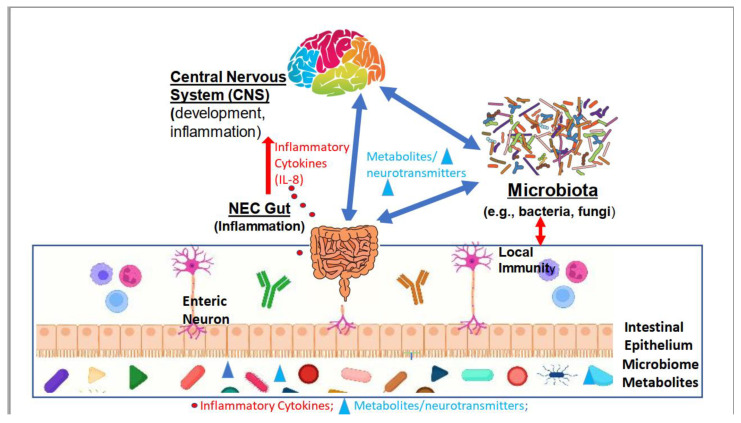
A working model of the gut microbiota–brain (GMB) axis in NEC. The interactions between the gut microbiota and brain are bidirectional. The balance between different microbiome taxa may result in dysbiosis, metabolite production, and inflammation that induce pathological changes in the gut and brain.

NEC remains a devastating illness with high morbidity and mortality. Increasingly, it is being recognized that the consequences of NEC extend beyond the gut. Long-term neurodevelopmental compromise impacts affected infants long after the NICU course. Pathogenesis of NEC is extremely complex and involves an interaction between the developing preterm infant and the microbiome (Figure 1). Immaturity of both the preterm gut and brain increases risk. The roles of microorganisms such as bacteria, viruses, fungi, and other microbes in the gut–brain axis of NEC are still incompletely understood. Studies on the early-life gut microbiome–brain axis will be critical for a better understanding of childhood health and diseases, as well as restorative methods for the prevention of NEC and treatment of diseases in adulthood. However, the unique promise of the microbiome is that it is modifiable in an individual child in a time frame relevant to influence their outcomes. For preterm infants, ongoing research offers the hope of microbiome-based biomarkers to identify preterm infants at risk for NEC and microbiome-based therapeutics to prevent and treat NEC as well as mitigate long-term consequences including neurodevelopmental injury.

## Data Availability

Not applicable.
